# Transport characteristics of salt ions in soil columns planted with *Tamarix chinensis* under different groundwater levels

**DOI:** 10.1371/journal.pone.0215138

**Published:** 2019-04-12

**Authors:** Ximei Zhao, Jiangbao Xia, Weifeng Chen, Yinping Chen, Ying Fang, Fanzhu Qu

**Affiliations:** 1 Shandong Key Laboratory of Eco-Environmental Science for the Yellow River Delta, Binzhou University, Binzhou, China; 2 College of Resources and Environment, Shandong Agriculture University, Tai’an, China; Shandong University, CHINA

## Abstract

The groundwater level is the main factor affecting the distribution of soil salinity and vegetation in the Yellow River Delta (YRD), China, but the response relationship between the spatial distribution of soil salt ions and the groundwater level in the soil-*Tamarix chinensis* system remains unclear. In order to investigate the patterns of soil salt ions responding to groundwater levels, in the ‘groundwater-soil-*T*. *chinensis*’ system. Soil columns planted with *T*. *chinensis*, a constructive species in the YRD, were taken as the study object, and six groundwater levels (0.3, 0.6, 0.9, 1.2, 1.5 and 1.8 m) were simulated under saline mineralization. The results demonstrated the following: As affected by groundwater, Na^+^ and Cl^-^ were the main ions in the *T*. *chinensis*-planted soil column, with a trend of decreasing first and then increasing by the increase of soil depth. However, the contents of K^+^ and NO_3_^-^ gradually decreased and CO_3_^2-^+HCO_3_^-^ gradually increased. As affected by groundwater evaporation, all the salt ions except CO_3_^2-^+HCO_3_^-^ exhibited different degrees of surface aggregation in the 0–20 cm layer. However, due to the impact of root uptake, the contents of the salt ions rapidly decreased in the root distribution layer (20–50 cm soil layer), which rendered a turning-point layer that was significantly lower than the surface soil layer; such decreases in ion contents showed a relatively large rate of variation. In the whole *T*. *chinensis*-planted soil column, with increasing groundwater level, the contents of Na^+^, Cl^-^, Ca^2+^, Mg^2+^, and NO_3_^-^ all tended to first decrease, then increase and decrease again, but the content of CO_3_^2-^+HCO_3_^-^ first decreased and then increased. Therefore, the 0.9 m groundwater level was the turning point at which the main salt ions underwent significant changes. The contents of Na^+^, Cl^-^, Ca^2+^ and Mg^2+^ in the *T*. *chinensis* planted soil column exhibited moderate variability (14.46%<CV<86.46%), with a relatively large degree of variability across the 20–50 cm root-concentrated distribution layer and the surface soil layer. However, the K^+^ content exhibited greater variability (CV>111.36%) at most groundwater level except less than 0.9 m. Therefore, planting *T*. *chinensis* could effectively reduce the accumulation of salt ions in the 20–50 cm soil layer with a concentrated root distribution, suggesting that the planting depth of *T*. *chinensis* should be greater than 20 cm under saline mineralization. This study can provide references for the control of soil secondary salinization and the management of *T*. *chinensis* seedling cultivation under saline mineralization.

## Introduction

Soil salinization is one of the most important land resources and environmental issues worldwide, and it urgently requires a solution [1–2]. With the increasing tension between population growth and natural resource management, the improvement and utilization of saline-alkali land resources have become the focus of research and attention in various countries around the world [3–5]. The Yellow River Delta (YRD) is one of the fastest land-forming estuarine deltas in China, as well as globally; and it is rich in natural resources and is an important reserve land resource [6]. However, this region is experiencing high groundwater evaporation and a large shortage of fresh water resources. It has frequent seasonal droughts and a fragile ecological environment, and the severe soil salinization has become a bottleneck, restricting the sustainable development of agriculture and forestry in this region. Under the influences of regional natural and human factors, saline soils in different bioclimatic zones have different occurrence characteristics and evolutionary patterns [7]. The salt composition and ion proportions of saline soils exhibit typical regional characteristics, and the salt accumulation and desalination processes are significantly different [8]. Particularly in the climate zone of arid deserts, salt-containing parent rocks and parent materials, active surface water, and groundwater recharge are the forces driving the formation of saline soils [7]. However, in the YRD, the groundwater depth is generally low due to seawater intrusion and sea level rise [9], and the shallow groundwater is the most sensitive factor and main source of water for the terrestrial saline-alkali vegetation during its key growth period along the muddy coast in this region [10–12]. The level and salinity of the groundwater control the contents and distributions of soil salts [13–15], which in turn affect the growth and development, distribution pattern, and community succession of the dominant vegetation in the YRD [16]. Furthermore, the vegetation growth and distribution are the main factors determining the recharge of and dynamic variations in groundwater [15,17]. Therefore, this study considered the water-salt coupling effect in the soil-plant system caused by groundwater variations to examine the distribution patterns of the main salt ions in the soil-plant system from the perspective of the groundwater level, which is of great scientific significance in terms of the effective control of soil secondary salinization, the efficient utilization of groundwater resources, and the cultivation and management of saline-alkali plants.

To address this urgent issue of saline soil types and their spatial differentiation, researchers in China and abroad have conducted a large number of studies using various methods including field surveys [18–19], model simulation [20], remote sensing [21–23] and simulation experiments [24–25] in combination with mathematical functions and statistical indices [26]. In saline-alkali land of arid areas or coastal saline-alkali land lacking freshwater resources, the transport of soil moisture, nutrients, salinity, and heat in the soil vertical profile varies greatly with the variation in soil depth. The groundwater evaporation and soil water capillary effect are the dominant factors determining soil salt variation [27]. Groundwater is the main factor affecting soil salt transport, accumulation and release, and the transport of soil water and salts are closely related to the groundwater level and salinity [11–12,15,28]. In our previous research, we found that the salt content of soil columns planted with *Tamarix chinensis* increased with increasing groundwater salinity under the same groundwater level [29], and the salinity altered the soil water and salt conditions, thus significantly affecting the growth, photosynthetic characteristics, and water consumption performance of *T*. *chinensis* [30]. When the groundwater level is deep, the groundwater evaporation rate and volume are small, and the soil does not undergo salinization, even if the evaporation-precipitation ratio is large. Only when the groundwater level reaches a certain critical depth can salts accumulate on the surface of the ground along with the capillary upward transport; salts migrate upward more readily under the capillary effect, especially when the groundwater salinity is high and the level is shallow [10]. The groundwater level has a great effect on soil water evaporation, and the soil water redistribution during during evaporation leads to the redistribution of salt ions in the soil profile [8–9]. The variation in the soil salt ion content is an important indicator affecting soil salinization, and the salt ions can more accurately determine the salinization type than salt content. Hence, investigating the transport characteristics of soil salt ions can provide a theoretical basis and technical support for the prevention of soil secondary salinization. To date, the study of soil water and salt characteristics in the YRD has mainly focused on aspects including soil salinization types and factor analysis [18,21], remote-sensing image analysis of soil salinity under macroscopic conditions [23], groundwater characteristics and the variability in soil water and salt contents [31], the effects of groundwater on the distribution of vegetation [32], and the interaction effects of water and salt [33–34]. Xia et al. [9] focused on the effects of soil water and salt contents on the variation in the Na^+^ content of *T*. *chinensis* by experimentally comparing soil columns planted with *T*. *chinensis* and bare land (CK). Unfortunately, the transport characteristics of salt ions (K^+^, Na^+^, Ca^2+^, Mg^2+^, Cl^-^, SO_4_^2-^, NO_3_^-^, CO_3_^2-^ and HCO_3_^-^) in soil columns planted with *T*. *chinensis* were not examined. Few reports have assessed the transport of soil salt ions in soil-plant systems under different groundwater levels and the differentiation patterns of salt ions. *T*. *chinensis* is a major vegetation species used in the restoration of the salinization area of the YRD, and it has a wide distribution. The species has a salt-secreting gland that allows the enrichment of salts in soils, and it can form a ‘salt island’ under plant clumps via biological effects [35], thereby playing an important role in improving the regional ecological environment and maintaining the stability of coastal ecosystems [9,12]. A preliminary survey found that *T*. *chinensis* degradation was determined by groundwater, soil moisture and salinity, soil nutrients and other environmental factors, but it is difficult to find plants of the same size in the wild. Therefore, the effects of the single factor of groundwater level on water, salt, salt ions, and planting *T*. *chinensis* under conditions of no surface water source and the same soil texture, plant and climatic factors remains unclear, but such an understanding is important for addressing the issues related to the transport characteristics of soil salt ions in the interaction effect of the groundwater-soil-plant system. Using soil columns planted with three-year-old *T*. *chinensis* seedlings as the study subject, saline mineralization was simulated under six groundwater levels, and the contents of the main cation ions including K^+^, Na^+^, Ca^2+^, and Mg^2+^ and the main anion ions including Cl^-^, SO_4_^2-^, NO_3_^-^, CO_3_^2-^, and HCO_3_^-^ in the soil profile under different groundwater levels and *T*. *chinensis* were measured and analysed to investigate the transport pattern responses of different salt ions in the soil profile to the groundwater level. These results can provide a theoretical basis and technical reference for the control of soil secondary salinization and for the management of water and salt in *T*. *chinensis* seedling cultivation in the YRD.

## Materials and methods

### Study area

The experiment was conducted at the research greenhouse of Binzhou University, Shandong Provincial Key Laboratory of Eco-Environmental Science for the YRD (117°58′57″E, 37°22′56″N), China. The groundwater in the YRD is affected by seawater, mainly NaCl, so to simulate saline water (20 g L^−1^), the groundwater was treated by adding sea salts from the YRD with a pH 7.4 and a salinity of 1.68%. The resulting groundwater salinity was 20.3 g L^−1^, and the corresponding ionic compositions are shown in Table 1. The soil samples used in this study were obtained from lowerland of the Yellow River in the YRD, belonging to alluvial soil with a pH of 7.54, bulk density of 1.32 g cm^-3^, field moisture capacity of 37.86% and salinity of 0.25%. Furthermore, the soil is a silty loam (5.76% clay, 47.66% silt, and 46.58% sand) with a fine and loose texture due to the alluviation of the Yellow River.

**Table 1:** Salt ions contents of groundwater under saline water.

K^+^ (μg·mL^-1^)	Na^+^ (mg·mL^-1^)	Ca^2+^ (μg·mL^-1^)	Mg^2+^ (μg·mL^-1^)	Cl^-^ (mg·mL^-1^)	CO_3_^2-^ (μg·mL^-1^)	HCO_3_^-^ (μg·mL^-1^)	SO_4_^2-^ (μg·mL^-1^)
18.17±3.15	7.10±0.23	55.88±4.61	95.11±7.18	11.52±1.83	16.92±1.85	158.32±15.62	376.07±34.28

### Experimental setup

The groundwater level of the YRD is shallow with an average depth of 1.1 m [21], which is greatly affected by distance from the sea. Because the groundwater level for growing *T*. *chinensis* ranges from 0.3 to 2.0 m [16], the following six groundwater levels were set: 0.3 m, 0.6 m, 0.9 m, 1.2 m, 1.5 m and 1.8 m; there were three replications for each water level.

**Fig 1:**
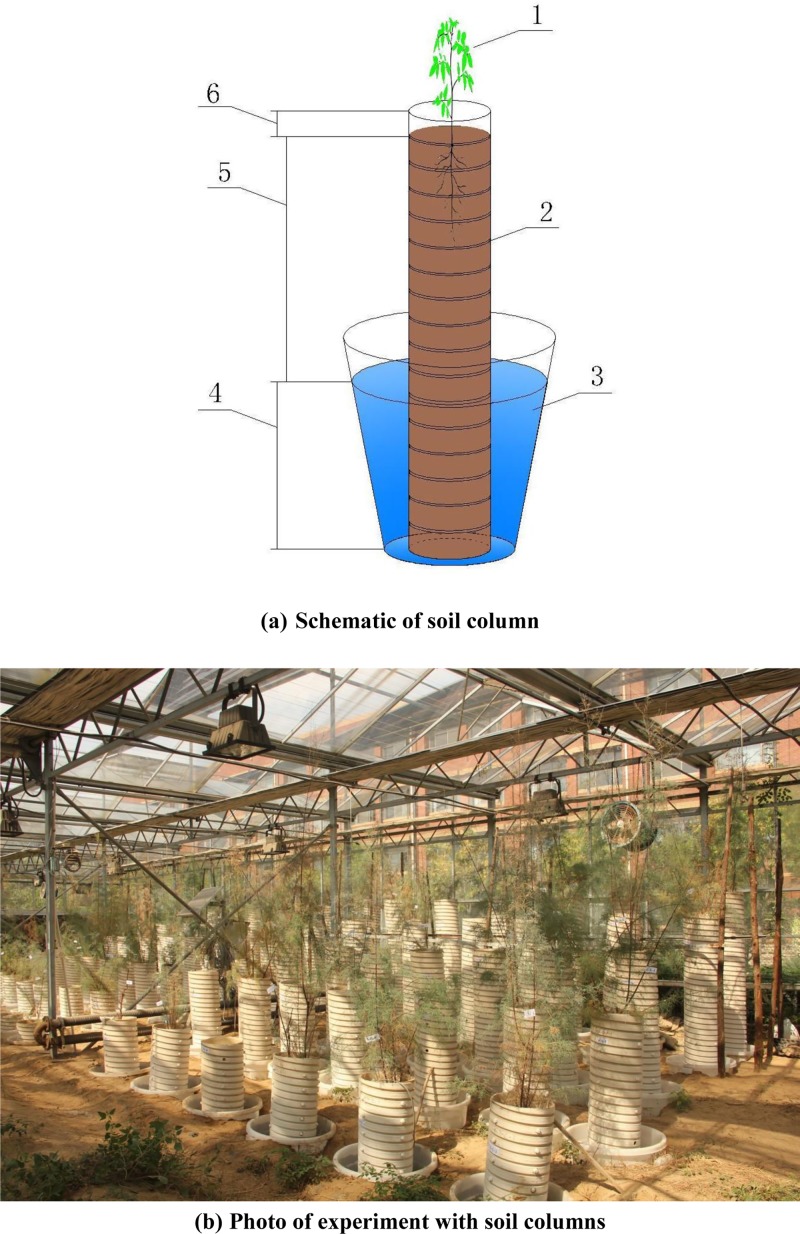
**Schematic diagram (a) and photo (b) of soil columns with planting *Tamarix chinensis*. (a)** Schematic of soil column; **(b)** Photo of experiment with soil columns **1,** *Tamarix chinensis* Lour; **2,** Soil; **3,** Groundwater; **4,** Flooded area at 0.55 m. **5,** Groundwater depths (0.3m-1.8 m); **6,** Interstice layer of 0.03 m.

The detailed experimental setup was as follows. In the research greenhouse, polyvinyl chloride (PVC) pipe (with an inner diameter of 30 cm) was used as the *T*. *chinensis* planting container, and a bucket (height × top diameter × bottom diameter = 0.70 m×0.57 m×0.45 m) was used as the groundwater simulation device. Buckets were buried in the soil by trenching to ensure a consistent groundwater temperature. According to the following equation, PVC pipe height = simulated groundwater level+depth of actual flooded layer (0.55 m)+depth of the top interstice layer (0.03 m), the PVC pipe was first cut into lengths of 0.88 m, 1.18 m, 1.48 m, 1.78 m, 2.08 m, and 2.38 m, and for each of the corresponding soil sampling depths, a 2.0 cm aperture was created around the PVC pipe as the soil sampling port and blocked with a plug. In the 0.55 m PVC pipe for the actual flooded area, four water inlets (each 1 cm in diameter) were generated every 10 cm along the pipe and blocked with permeable cloth, and an anti-filter layer was laid to ensure the simulated groundwater could enter the soil column from the bottom and the surrounding inlets. Next, soil columns were filled with soil layer by layer, with each layer consisting of 20 cm, according to the soil bulk density, and the soil inter-layers were then compacted. Finally, three-year-old *T*. *chinensis* seedlings (1.3 cm in root diameter and 60 cm in height) with uniform growth were planted in the PVC pipes. Three plants were initially planted in each container; groundwater level control was simulated after one month of normal management, and only one surviving seedling remained for further study. The diagram of the simulation design and the real image of the soil columns planted with *T*. *chinensis* are shown in Fig 1.

### Sampling and analytical procedures

The experiment began on March 2015 after preparing the experimental device. The soil samples were collected, and the salt ion parameters were measured in June 2015. Based on the simulation experiment and the pertinent literature [16,23], the soil profile sampling interval was designed as follows: each soil layer was 10 cm if the groundwater level was 30–60 cm; each soil layer was 20 cm, including a 0–10 cm surface soil layer, if the groundwater level was 90–120 cm; and if the groundwater level exceeded 120 cm, the soil layer was equal to 30 cm including a 0–10 cm surface soil layer. Three replications were performed for each soil layer.

The soil samples were transported to the laboratory, air-dried for two weeks, evenly mixed and crushed, and then sieved through a 2.0 mm screen. The salinity, pH and electrical conductivity (EC) of the groundwater were measured in situ by a multi-parameter water quality analyser (Horiba U52, Japan), and the water-soluble salt in the soil was extracted according to the forestry industry standards of the People's Republic of China (LY/T 1251–1999). Soil salt anions (Cl^-^, SO_4_^2-^, and NO_3_^-^) were analysed by ionic chromatography (Dionex IC 2000, America), and cations (K^+^, Na^+^, Ca^2+^, and Mg^2+^) were analysed by flame atomic absorption spectrometry (Shimadzu AA 6800, Japan). During the process of determination, 1% CsNO_3_ was added in solution in the process of determination in order to prevent the K^+^ and Na^+^ from ionizing, and 5% LaCl_2_ was added to prevent the Ca^2+^ or Mg^2+^ and phosphate producing precipitate. CO_3_^2-^ and HCO_3_^-^ were determined by standard titration with sulfuric acid.

### Statistical analysis

The experimental data were processed and plotted with Excel 2010 (Microsoft Corp. Redmond, WA, USA), while one-way ANOVA was performed using the Statistical Analysis System 9.0 (SAS Institute Inc. Cary, NC, USA), in order to identify the differences of the average salt ions content in the *T*. *chinensis*-planted soil column among the six groundwater levels. Differences were considered to be significant if *P<*0.05.

## Results and analysis

### Effects of different groundwater levels on salt cations in the soil profile

#### Variation in the Na^+^ content in the soil profile

**Fig 2:**
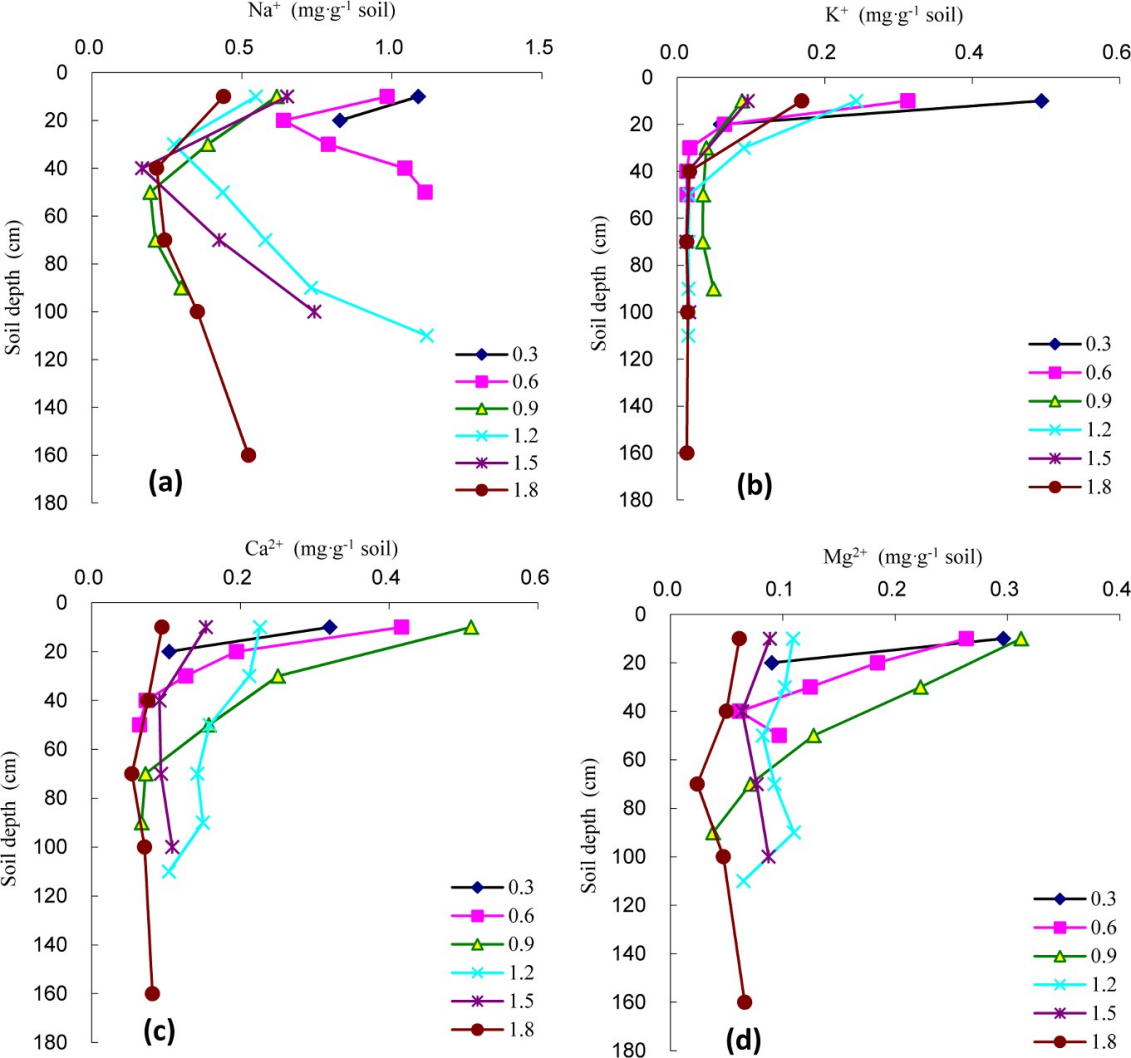
Changes of soil salt cation under different groundwater level in soil profiles. **(a)** Na^+^; **(b)** K^+^; **(c)** Ca^2+^; **(d)** Mg^2+^.

As shown in Fig 2A, the Na^+^ content decreased with the increase in soil depth under the 0.3 m groundwater level, but no significant differences were observed between the surface soil layer and the deep soil layer (*P*>0.05), with a coefficient of variation (CV) of only 19.32%. The Na^+^ contents under the other groundwater levels all first decreased and then increased with the increase in soil depth, and they showed different degrees of surface aggregation. The turning point at which the lowest Na^+^ content occurred under each groundwater level was at a soil depth of 20–50 cm, below which the Na^+^ content gradually increased with increasing soil depth.

**Fig 3:**
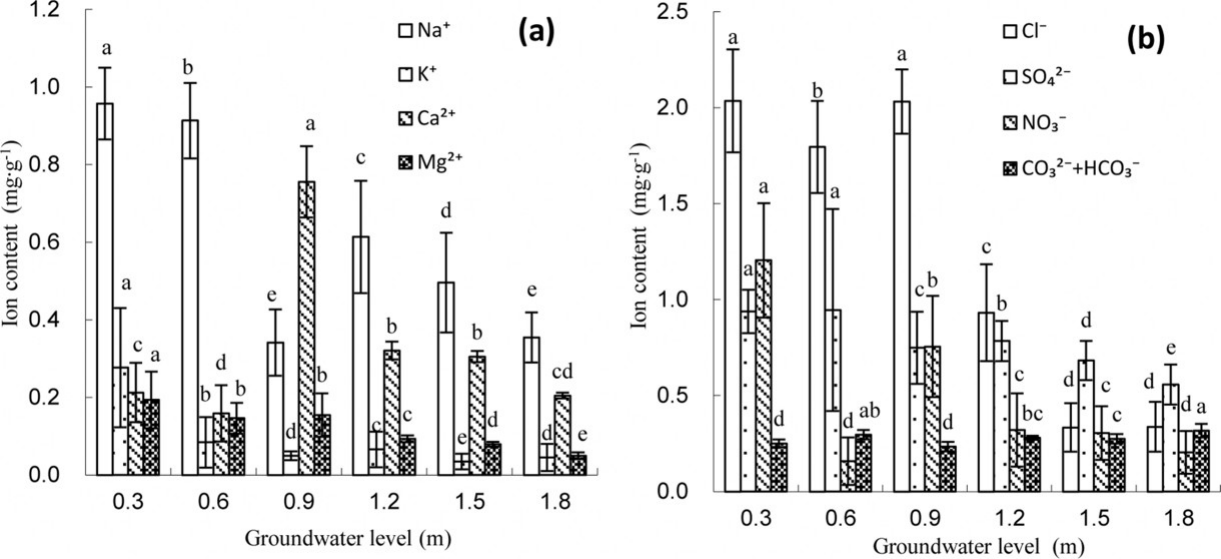
Content of salt ion under different groundwater level in soil columns. **(a)** Salt cation; **(b)** Salt anion.

With an increase in the groundwater level, the Na^+^ content in the surface soil layer gradually decreased overall, with the Na^+^ content descending with the groundwater level in the following order: 0.3 m>0.6 m>1.5 m>0.9 m>1.2 m>1.8 m. The Na^+^ contents in the surface layer under soil groundwater levels of 0.3 m, 0.6 m, 0.9 m, 1.2 m, and 1.5 m were, respectively, 2.48, 2.24, 1.40, 1.25, and 1.48 times those under the 1.8 m groundwater level (0.439 mg g^-1^); no significant differences were detected between the Na^+^ content in the surface soil layer and the groundwater levels from 0.9 m to 1.2 m (*P*>0.05). For the entire soil column (Fig 3A), the average soil Na^+^ content tended to first decrease and then increase with the increasing groundwater level, behaving similarly to the variation in Na^+^ in the surface soil layer. The average Na^+^ content in the *T*. *chinensis*-planted soil column under each of the various groundwater levels ranged from 0.341 to 0.957 mg g^-1^, rendering Na^+^ the main salt cation; the CV of the Na^+^ contents across soil layers in the soil column under each of the various groundwater levels ranged from 19.39% to 51.72%. The lowest average Na^+^ content in the soil column occurred at a groundwater level of 0.9 m, rendering 0.9 m the turning-point groundwater level.

#### Variation in the K^+^ content in the soil profile

As shown in Fig 2B, the soil K^+^ content gradually decreased with increasing soil depth under each groundwater level, exhibiting a rapid decrease in the 20–50 cm soil layer and a steady variation below. The K^+^ contents in the surface soil layer and the other soil layers were significantly different (*P<*0.05), exhibiting a pronounced surface aggregation pattern.

With an increasing groundwater level, the K^**+**^ content in the surface soil layer displayed a “W” pattern, with its variation pattern descending with the groundwater level in the following order: 0.3 m>0.6 m>1.2 m>1.8 m>1.5 m>0.9 m; the lowest K^+^ content was observed under the 0.9 m groundwater level, which was 0.088 mg g^-1^. The K^+^ contents in the surface layer under soil groundwater levels of 0.3 m, 0.6 m, 1.2 m, 1.5 m, and 1.8 m were, respectively, 5.62, 3.55, 2.76, 1.09, and 1.92 times those under the 0.9 m groundwater level (0.088 mg g^-1^). As shown in Fig 3B, the average K^+^ content in the *T*. *chinensis*-planted soil column showed a consistent tendency to vary with the K^+^ content in the surface soil layer with an increasing groundwater level, but the ranges in variation were relatively large in the surface soil layer, middle soil layer, and deep soil layers. Under the groundwater level of 0.9 m, the CV of the K^+^ contents across the various soil layers was lowest at 45.67% in the *T*. *chinensis*-planted soil column, but under the other groundwater levels, the CVs could reach as high as 111.36–154.29%.

#### Variations in the Ca^2+^ and Mg^2+^ contents in the soil profile

As shown in Fig 2C and 2D, the variations in Ca^2+^ and Mg^2+^ were almost equivalent under the same groundwater level, and both exhibited different degrees of surface aggregation. Under groundwater levels of 0.3 m, 0.6 m, 0.9 m, and 1.2 m, the contents of Ca^2+^ and Mg^2+^ in the soil profile decreased with increasing soil depth, with the rate of variations exhibiting a fast-slow decreasing trend. However, under deep groundwater levels of 1.5 m and 1.8 m, the Ca^2+^ and Mg^2+^ contents first decreased and then increased with increasing soil depth, with the middle soil layer (40–70 cm) representing the turning-point.

With an increasing groundwater level, the Ca^2+^ and Mg^2+^ contents in the surface layer first increased and then decreased, and the highest contents occurred under the 0.9 m level and were 0.510 mg g^-1^ and 0.312 mg g^-1^, respectively, representing 5.39 and 5.08 times the Ca^2+^ and Mg^2+^ contents in the surface soil layer under the deep groundwater level of 1.8 m. As shown in Fig 3A, the average contents of Ca^2+^ and Mg^2+^ in the *T*. *chinensis*-planted soil column both tended to first decrease, then increase, and then decrease again, with the middle groundwater level of 0.9 m representing the turning point where the second decrease occurred. The Ca^2+^ content in the *T*. *chinensis*-planted soil column ranged from 0.076 to 0.212 mg g^-1^, and the CV ranged from 19.63% to 86.47%. The Mg^2+^ content ranged from 0.050 to 0.193 mg g^-1^, and the CV ranged from 14.47%–72.97%.

### Effect of different groundwater levels on salt anions in the soil profile

#### Variation in the Cl^-^ content in the soil profile

**Fig 4:**
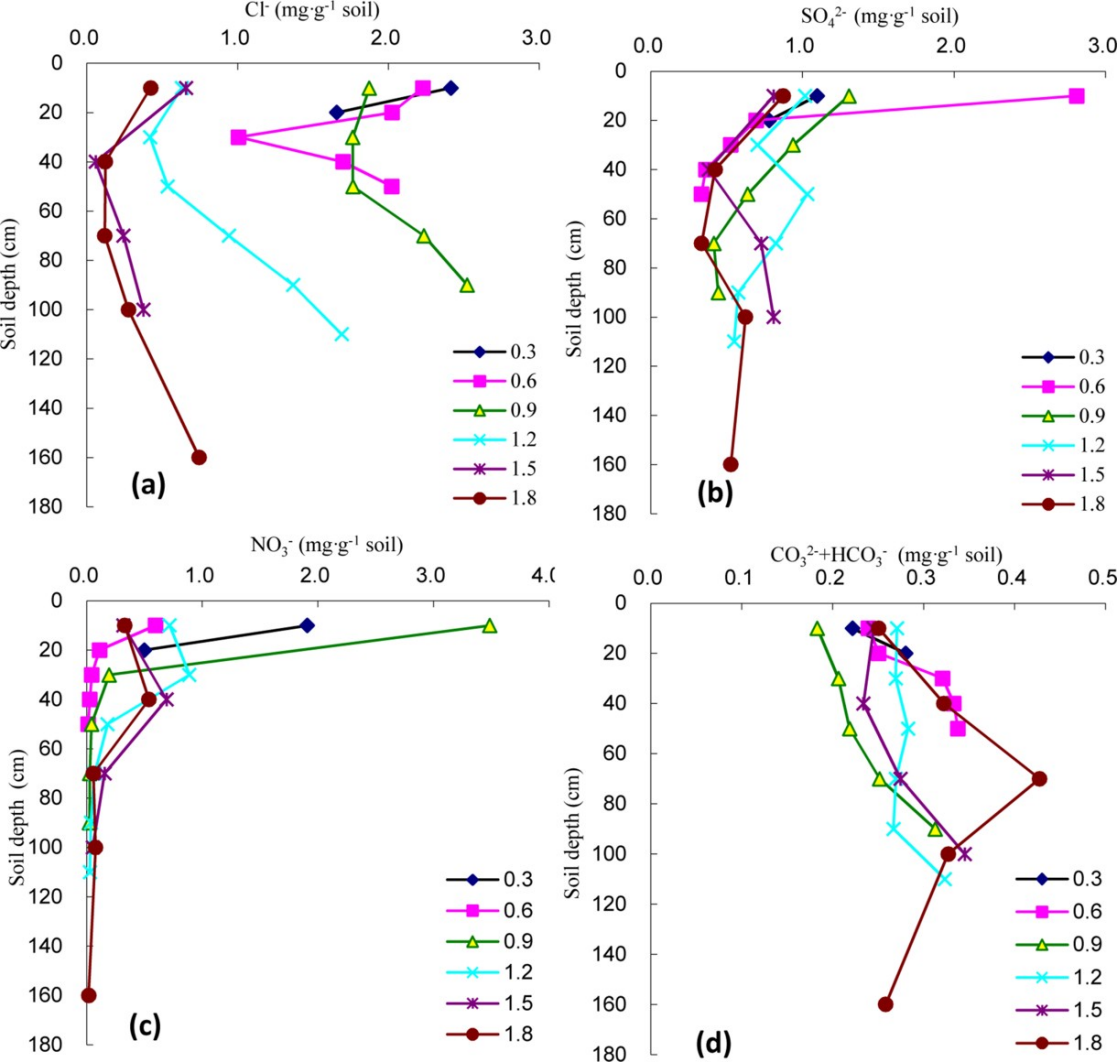
Changes of soil salt anion under different groundwater level in soil profiles. **(a)** Cl^-^; **(b)** SO_4_^2-^; **(c)** NO_3_^-^; **(d)** CO_3_^2-^+HCO_3_^-^.

As shown in Fig 2A and Fig 4A, the Cl^-^ content displayed a similar variation in Na^+^ content under different groundwater levels. Although the soil Cl^-^ content showed a significantly negative correlation with soil depth under the 0.3 m groundwater level (*P<*0.05), those under the other groundwater levels first decreased and then increased with the lowest values in the 30–50 cm soil layer, exhibiting an obvious surface aggregation pattern.

With an increasing groundwater level, the Cl^-^ content in the surface soil layer gradually declined. No significant difference was detected among the Cl^-^ contents in the surface soil layer under the three groundwater levels of 0.3 m, 0.6 m, and 0.9 m or under the levels of 1.2 m, 1.5 m, and 1.8 m (*P*>0.05), but there was a significant difference between 0.9 m and 1.2 m (*P<*0.05). Compared with the Cl^-^ content in the surface soil layer under the 0.3 m groundwater level (2.415 mg g^-1^), those under the 0.6 m, 0.9 m, 1.2 m, 1.5 m, and 1.8 m groundwater levels decreased by 7.72%, 22.44%, 73.87%, 72.74%, and 82.46%, respectively. Under each of the various groundwater levels, the average Cl^-^ contents in the *T*. *chinensis*-planted soil column and the CVs ranged from 0.334 to 2.035 mg g^-1^ and 16.53% to 77.22%, respectively; Cl^-^ was the main anion responsible for soil salinity. With an increasing groundwater level, the average Cl^-^ content first decreased, then increased, and then decreased again, with the highest content detected under the 0.9 m groundwater level. When the groundwater levels exceeded 0.9 m, the average Cl^-^ contents in the *T*. *chinensis*-planted soil column differed significantly (*P<*0.05) and declined rapidly (Fig 3B).

#### Variation in the SO_4_^2-^ content in the soil profile

As shown in Fig 4B, SO_4_^2-^ in the soil profile exhibited a decreasing trend with an increasing soil depth under groundwater levels of 0.3 m, 0.6 m, and 0.9 m, while it tended to rapidly decline followed by a gradual increase under deep groundwater levels of 1.5 m and 1.8 m. This profile was similar to those observed for the soil salt cations Ca^2+^ and Mg^2+^.

With an increasing groundwater level, the SO_4_^2-^ content in the surface soil layer first increased and then decreased, reaching the highest level under a level of 0.6 m and exhibiting an obvious pattern of surface aggregation, similarly to the contents of Ca^2+^ and Mg^2+^. The SO_4_^2-^ contents in the surface soil layer under groundwater levels of 0.3 m, 0.9 m, 1.2 m, 1.5 m, and 1.8 m were 39.07%, 46.54%, 36.26%, 28.86%, and 31.11% of those under the 0.6 m groundwater level, respectively. As shown in Fig 3B, the average SO_4_^2-^ content in the *T*. *chinensis*-planted soil column was highest under the 0.6 m groundwater level at 0.945 mg g^-1^. This was not significantly different from that under the 0.3 m groundwater level (*P*>0.05), but it differed significantly from other groundwater levels (*P<*0.05). In the *T*. *chinensis*-planted soil column, the SO_4_^2-^ content basically showed a mild ‘M’ pattern with increasing groundwater level, with the 0.9 m groundwater level as the turning point at which the average SO_4_^2-^ content significantly decreased.

#### Variation in the NO_3_^-^ content in the soil profile

As shown in Fig 4C, with an increasing soil depth and under groundwater levels of 0.3 m, 0.6 m and 0.9 m, the NO_3_^-^ contents in the soil profile all tended to rapidly decline in the 30 cm soil layer and then decline more slowly, which was consistent with the variation in the content of the salt cation K^+^. For example, with an increasing soil depth under the 0.9 m groundwater level, the NO_3_^-^ contents in the other four soil layers (30–90 cm) decreased by 94.39%, 98.77%, 99.19%, and 99.27%, respectively, compared to that in the surface soil layer. Under the groundwater levels of 1.2 m, 1.5 m, and 1.8 m, the NO_3_^-^ contents in the soil all first increased and then decreased with increasing soil depth, with the highest values detected in the 30–40 cm soil layer.

With an increasing groundwater level, the average NO_3_^-^ contents in the *T*. *chinensis*-planted soil column all tended to first decrease, then increase, and decrease again, which was consistent with the pattern in the surface soil layer. Under the 0.9 m groundwater level, the highest NO_3_^-^ content was detected in the surface soil layer at 3.485 mg g^-1^, which was 1.83 and 5.85 times that under the groundwater levels of 0.3 m and 0.6 m, respectively (*P<*0.05). When the groundwater levels were greater than 1.2 m, the NO_3_^-^ contents exhibited a small range of variation within the *T*. *chinensis*-planted soil column, yet the CVs were still greater than 100%.

#### Variation in the CO_3_^2-^+HCO_3_^-^ content in the soil profile

As shown in Fig 4D, the CO_3_^2-^ +HCO_3_^-^ content first increased and then decreased in the soil profile with increasing soil depth under the 1.8 m groundwater level, and it exhibited an increasing trend in the soil profile under the other groundwater levels. In particular, with an increasing soil depth under the 0.9 m groundwater level, the CO_3_^2-^ +HCO_3_^-^ contents in the other four soil layers were, respectively, 58.54%, 66.02%, 69.92%, and 80.49% of that in the bottom soil layer.

The CO_3_^2-^ +HCO_3_^-^ content in the surface soil layer and in the *T*. *chinensis*-planted soil column first decreased and then increased with an increasing groundwater level. The lowest contents in the surface soil layer and the *T*. *chinensis*-planted soil column were both under the 0.9 m groundwater level and were 0.183 mg g^-1^ and 0.234 mg g^-1^, respectively. The average CO_3_^2-^ +HCO_3_^-^ contents in the *T*. *chinensis*-planted soil column ranged from 0.234 to 0.317 mg g^-1^, and the CVs ranged from 7.77% to 22.41%.

## Discussion

### Interaction effect between the vertical distribution of soil salt ions and the groundwater level

The degree of salt accumulation in soil is directly related to atmospheric evaporation, soil lithology, groundwater level, and groundwater mineralization; particularly, under conditions of no irrigation, no rainfall, a given atmospheric evaporation capacity, and a given soil lithology, soil water and salt transport are closely related to the groundwater level [16, 36]. Previous studies have shown that groundwater is the main factor affecting soil salinization, and the degree of salt accumulation in soil is mainly dependent on the groundwater level and salinity [31]. Under strong evaporation, soil salts are dissolved in the groundwater, and by using water rising through capillary action as the carrier, they gradually move upward to accumulate in the surface layer. When the rising water enters the atmosphere through the diffusion of water vapour, the salts remain in the shallow soil layer. In the YRD, which has a large evaporation-precipitation ratio and shallow groundwater level, groundwater evaporation is the reason for secondary salinization [34]. During the processes of water evaporation and the upward movement of capillary water, water and salt are redistributed, and the soluble ions carried in the groundwater (or soil solution) gradually aggregate towards the surface soil layer [37]. When water evaporates and enters the atmosphere, the soluble salt ions are retained in different soil layers based on the different rates of ion transport, which results in the redistribution of soil salt ions, which vary greatly under different groundwater levels [8,38–39].

The results showed that the variation in the Na^+^ content was similar to that in the Cl^-^ content in the vertical soil profile under different groundwater levels. Under the 0.3 m level, the contents of Na^+^ and Cl^-^ decreased with increasing soil depth; in contrast, they first decreased and then increased with an increasing soil depth under the other groundwater levels, exhibiting an obvious pattern of surface aggregation. The contents of both ions tended to mimic the distribution of soil salinity [34]. The above variations were mainly due to the influence of seawater intrusion. In the YRD, NaCl is a main component of the groundwater, which has a high degree of mineralization. Both Na^+^ and Cl^-^ are weakly adsorbed by soil colloids, and they are both monovalent charge carriers with a strong migration ability to transport along with water and thus highly correlated ions [39–40] with a coordinated migration relationship [41]. Therefore, under different groundwater levels, the vertical variation patterns of Na^+^ and Cl^-^ were similar. A study by Guo et al. found that the Na^+^ and Cl^-^ contents under various groundwater levels showed a gradually decreasing trend with an increasing soil depth and a significant pattern of surface aggregation[42], which was consistent with the variation pattern in the middle and shallow soil layers (0–20 cm) in the present study. However, in this study, the contents of Na^+^ and Cl^-^ exhibited an increasing trend below the 20–50 cm soil layer that was mainly due to the shallow groundwater that enabled a continuous supply of salt ions to be transported through water. In addition, the deep-layer soil was in the saturated aquifer, resulting in high Na^+^ and Cl^-^ contents.

Under the same groundwater level in this study, the variations in Ca^2+^, Mg^2+^ and SO_4_^2-^ were basically equivalent, yet their rates of variation was not as drastic as those of Na^+^ and Cl^-^. The correlation analysis of the ions also suggested that Ca^2+^ and Mg^2+^ were most strongly related, and they are usually grouped into the same category in studies of ion transport [39,43]. Due to the effects of ionic charge, hydration radius, ion concentration, and other characteristics, Ca^2+^, Mg^2+^, and SO_4_^2-^ had a strong capacity to be absorbed by soil colloids and were minimally influenced by the movement of irrigation water. Hence, during the upward and downward movement of salt, the degree of activity of the chemicals showed a descending order of chloride>sulfate>carbonate [44]. Under the groundwater levels of 1.5 m and 1.8 m, the Ca^2+^, Mg^2+^, and SO_4_^2-^ contents first decreased and then increased with an increasing soil depth, and they were negatively correlated with soil depth under the other groundwater levels, which was basically consistent with the results obtained for the saline soils in Gansu Province by Guo et al.[42] and Yang et al.[45].

Under different groundwater levels, both the K^+^ and NO_3_^-^ contents decreased with an increasing soil depth, with drastic variations observed in the surface layer and mild variations in the other soil layers. When the groundwater level was greater than 1.2 m, the highest soil NO_3_^-^ content did not occur in the surface soil layer but concentrated at a depth of 30–40 cm because NO_3_^-^ was not easily adsorbed by soil colloids and moved with water. The groundwater in the soil partly rises with capillary water, which is affected by soil bulk density, clay content, groundwater salinity, groundwater depth and infiltration time, etc. and partly by evaporation into the atmosphere or lifting of the plant by the roots. In this study, the soil was obtained from the Lower Yellow River in the YRD and was silty loam (5.76% clay, 47.66% silt, 46.58% sand) with a fine and loose texture due to alluvial of the Yellow River, and the soil texture affected the height of the capillary rise. Xia et al. also found that the groundwater could supply a sufficient aeration zone through capillary effects to maintain the wetting of the surface soil layer when the groundwater level was shallow (less than 0.9 m) [9]. With an increasing groundwater level, the distance that the groundwater moved upward to the surface and shallow soil layers increased, and due to the weakening of gravity and the capillary effect, it exceeded the critical capillary depth, leading to the formation of a dry and water-deficient surface soil layer. These findings are consistent with the conclusions of the present study indicating that the highest NO_3_^-^ content occurred in the 30–40 cm soil layer when the groundwater level was greater than 1.2 m.

The CO_3_^2-^+HCO_3_^-^ contents in the soil profile increased with increasing soil depth but with a small range of variation range between soil layers, and the carbonate ions showed relatively stable transport, which was consistent with results obtained for the cracked alkaline soil in the Yinbei District of Ningxia Province [41]. The patterns of variation in soil carbonates in Bohai Rim were opposite those of other salt ions, and the HCO_3_^-^ content was negatively correlated with the contents of other salt ions [46]. The HCO_3_^-^ content in the surface soil layer of the simulated soil columns decreased with an increasing groundwater level, and bicarbonate had the smallest solubility and was the first to be precipitated when transported with water [42]. In a study simulating the arid soil in Xinjiang, China [27] and water transport in soil columns planted with *T*. *chinensis* in the YRD [34], it was found that the water content in the soil profile increased with an increasing soil depth. The surface aggregation ability of Na^+^ and Ca^2+^ in the soil increased, and due to the hydrolysis of Na^+^, the amount of OH^-^ increased. Due to the reaction of HCO_3_^-^ and OH^-^ to generate CO_3_^2-^ and H_2_O, the surface-aggregated CO_3_^2-^ and Ca^2+^ proceeded to form CaCO_3_ precipitate [27], resulting in a decreased HCO_3_^-^ content in the surface soil layer. This result was consistent with the variation pattern of the CO_3_^2-^+HCO_3_^-^ content observed in the present study.

### Factors impacting soil salt surface aggregation and the abrupt variation in the salt layer in the soil profile

Due to the effects of groundwater evaporation, the soil salinity exhibited different degrees of surface aggregation. In this study, all the other salt ions except CO_3_^2-^+HCO_3_^-^ showed different degrees of surface aggregation in the surface soil layer. When the groundwater level was shallow or the groundwater salinity was high, the salt contents in the soil profile were significantly affected by the groundwater level; a lower groundwater level resulted in a higher salt content in the soil profile [47]. Driven by evaporation, groundwater would move upward by capillary action to cause salt to accumulate in the surface soil layer [8,34,48], with Na^+^ and Cl^-^ accumulating most rapidly and exhibiting the highest concentration. Ye et al. also found that the accumulation of SO_4_^2-^ and Cl^-^ mainly occurred in the shallow soil layer, and the variation in SO_4_^2-^ in the deep soil layer was not obvious [20]. Na^+^ and Cl^-^ are the main ions in the groundwater and saline soil in the YRD, and since they are easily washed away by water, the salt content in the surface soil layer can be controlled by freshwater irrigation.

Salt could aggregate in the surface soil layer, but it can be caused by groundwater evaporation [15,48]. However, after selective absorption of soil salts by plant roots, the turning points for salts and salt ions (where the lowest contents occurred) occurred, and salts and salt ions were transported to stems and leaves [49]. *Tamarix chinensis* is a salt-secreting plant that is rich in salt-secreting glands, so salts can be secreted throughout the stems and leaves and then be driven into the surface soil layer by gravity, thereby producing salt surface aggregation [35,48]. The salt pump plays a role in the gain and loss of salts in the rhizosphere of desert halophytes and promotes salt transport to aboveground tissues.

At soil depths ranging from 20–50 cm, the contents of Na^+^, Cl^-^, K^+^, Ca^2+^, Mg^2+^, SO_4_^2-^, and NO_3_^-^ showed a rapid decline at the turning points. The contents of ions in the deeper soil layers were all clearly lower than those in the surface soil layer, and the range of variation was quite large, similar to that observed in other related studies [20,41,45]. The differences in the rate of salt ion migration and the salt absorption by roots of different biomass in various soil layers were the main factors underlying the variations in the salt contents in the vertical soil profile [35]. Song et al. found that the maximum depth range of *T*. *chinensis* is approximately 46.6–82.0 cm near the Yiqianer management station in Dongying City, Shandong Province, and the roots of the species are more distributed in shallow soil, where they account for more than 70% of the total biomass in the 0–30 cm soil depth [50]. Zhao et al. also found that the root biomass of *T*. *chinensis* was mainly distributed in the upper and middle soil layer in the one to three-year-old *T*. *chinensis* plantations in the YRD; the 2nd-instar roots were mostly in the 20–60 cm soil layer while the 3rd instar roots were primarily in the 40 cm soil layer [51]. Due to the influence of root growth and plant water absorption, there were some differences in the abrupt turning points for different salt ions [8,52–53]. The NO_3_^-^ content significantly decreased from the surface layer to the 40–60 cm soil layer or showed the lowest content in the root concentrated-distribution layer [20], but it approached a steady content in the deeper soil layer. However, the contents of Na^+^, Cl^-^, Ca^2+^, Mg^2+^, and SO_4_^2-^ first decreased and then increased with increasing soil depth and exhibited a significant decrease in the soil layer from 20–40 cm [45], which was consistent with the results of the present study.

### Vertical variability of soil salt ions affected by the groundwater level

The CV can reflect the degree of dispersion of random variables; CV<10% generally denotes weak variability; 10%<CV<100% denotes moderate variability; and CV>100% denotes strong variability [54,55]. The CV values of salt ions in the *T*. *chinensis*-planted soil column can, to a certain extent, reflect the distribution characteristics of different ions in the vertical soil profile, as well as differences between different ions in the rate of transport along with water and salt.

The contents of Na^+^, Cl^-^, Ca^2+^, and Mg^2+^ exhibited moderate variabilities in the *T*. *chinensis*-planted soil column. With an increasing groundwater level, the CVs for the Na^+^ and Cl^-^ contents across various soil layers in the soil column tended to first increase and then decrease. In addition, the degree of variability throughout the 20–50 cm root concentrate-distribution layer and surface soil layer and across the 20–50 cm root concentrate-distribution layer and deep soil layer were higher than that across the surface soil layer and the deep soil layer, which was mainly due to the surface aggregation of Na^+^ and Cl^-^ and the absorption of these salt ions by roots. Under low groundwater levels, the Ca^2+^ and Mg^2+^ contents in the *T*. *chinensis*-planted soil column showed a relatively large variability; when the groundwater level wasmore than 1.2 m, the variability in the soil column decreased.

With an increasing groundwater level, the degree variability in SO_4_^2-^ first increased and then decreased in the *T*. *chinensis*-planted soil column. Excluding the CV>100% in the soil column that indicated strong variability under the 0.6 m groundwater level, the SO_4_^2-^ content showed moderate variabilities under the other groundwater levels, which was attributed to the strong surface aggregation caused by the accompanying movement of this salt ion with water and salt under the 0.6 m groundwater level. Additionally, under various groundwater levels, the variabilities of the K^+^ and NO_3_^-^ contents in the *T*. *chinensis*-planted soil column were mainly due to the strong variability in the surface soil layer and root concentrated-distribution layer, which was jointly caused by the surface aggregating nature of these two ions, ion adsorption characteristics of the soil, ionic radius, and root absorption [8,52]. Furthermore, the CO_3_^2-^+HCO_3_^-^ content in the *T*. *chinensis*-planted soil column had a CV ranging from 7.77% to 22.41%, indicating little variability, which was consistent with the results reported by Guo et al. and Zhang et al. for saline soil in a semi-arid region [42,56].

## Conclusions

Under different groundwater levels, the variation trends of soil salt ions were quite different with increasing soil depth, and some ions showed abrupt variation points. With an increasing soil depth, the Na^+^ and Cl^-^ contents first decreased and then increased, exhibiting a pronounced surface aggregation pattern, and K^+^ and NO_3_^-^ gradually declined, with a drastic variation in the surface soil layer and a small variation in the bottom soil layer. The Ca^2+^, Mg^2+^, and SO_4_^2-^ contents were negatively correlated with soil depth except the groundwater level of 1.5 m and 1.8 m. The CO_3_^2-^+HCO_3_^-^ content gradually increased.

Planting *T*. *chinensis* could reduce the accumulation of soil salt ions in the soil layer with a concentrated root distribution. The main salt ions contents in the root distribution layer (20–50 cm soil layer) significantly decreased, rendering an abrupt variation in the soil profile.

The contents of salt ions in the surface soil layer were more significantly affected by the groundwater level than those in the other soil layers, and there were large differences in content variation of different salt ions with changes in the level of groundwater. Due to groundwater evaporation, salt ions in the surface soil layer showed different degrees of surface aggregation, except CO_3_^2-^+HCO_3_^-^ first decreased and then increased with an increasing groundwater level. To plant *T*. *chinensis*, it is recommended that the surface soil layer be avoided, and a depth below the top 20 cm soil layer is considered suitable.

Under saline mineralization, the average contents of salt ions in the whole soil column were closely related to the groundwater level, but large differences were observed among various ions. With an increasing groundwater level, the average contents of Na^+^, Cl^-^, Ca^2+^, Mg^2+^, and NO_3_^-^ of the whole soil column first decreased, then increased, and decreased again; and the 0.9 m groundwater level was the turning point at which the contents of these ions exhibited significant variations. However, the CO_3_^2-^+HCO_3_^-^ with small variation across soil layers. The Na^+^, Cl^-^, Ca^2+^ and Mg^2+^ contents in the *T*. *chinensis*-planted soil column showed moderate variabilities, but K^+^ with strong variability with groundwater level over 0.9 m.

## Supporting information

S1 Dataset Data for this manuscript (Data of the manuscript.xlsx). (XLSX)
